# Clonal and atypical *Toxoplasma* strain differences in virulence vary with mouse sub-species

**DOI:** 10.1016/j.ijpara.2018.08.007

**Published:** 2019-01

**Authors:** Musa A. Hassan, Aude-Anais Olijnik, Eva-Maria Frickel, Jeroen P. Saeij

**Affiliations:** a Division of Infection and Immunity, The Roslin Institute, College of Medicine and Veterinary Medicine, The University of Edinburgh, Edinburgh, UK; b Centre for Tropical Livestock Health and Genetics, The University of Edinburgh, Edinburgh, UK; c Host-Toxoplasma Interaction Laboratory, The Francis Crick Institute, London, UK; d Department of Pathology, Microbiology and Immunology, University of California, Davis, Davis, CA 95616, USA

**Keywords:** *Toxoplasma gondii*, Mouse sub-species, Wild-derived mice, Host–parasite interactions, ROP5, ROP18

## Abstract

The severe virulence of *Toxoplasma gondii* in classical laboratory inbred mouse strains contradicts the hypothesis that house mice (*Mus musculus*) are the most important intermediate hosts for its transmission and evolution because death of the mouse before parasite transmission equals death of the parasite. However, the classical laboratory inbred mouse strains (*Mus musculus domesticus*), commonly used to test *Toxoplasma* strain differences in virulence, do not capture the genetic diversity within *Mus musculus*. Thus, it is possible that *Toxoplasma* strains that are severely virulent in laboratory inbred mice are avirulent in some other mouse sub-species. Here, we present insight into the responses of individual mouse strains, representing strains of the genetically divergent M*us musculus musculus*, *Mus musculus castaneus* and *Mus musculus domesticus*, to infection with individual clonal and atypical *Toxoplasma* strains. We observed that, unlike *M. m. domesticus*, *M. m. musculus* and *M. m. castaneus* are resistant to the clonal *Toxoplasma* strains. For *M. m. musculus*, we show that this is due to a locus on chromosome 11 that includes the genes that encode the interferon gamma (IFNG)-inducible immunity-related GTPases (Irgs) that can kill the parasite by localising and subsequently vesiculating the parasitophorous vacuole membrane. However, despite the localization of known effector Irgs to the *Toxoplasma* parasitophorous vacuole membrane, we observed that some atypical *Toxoplasma* strains are virulent in all the mouse strains tested. The virulence of these atypical strains in *M. m. musculus* could not be attributed to individual rhoptry protein 5 (ROP5) alleles, a secreted parasite pseudokinase that antagonises the canonical effector Irgs and is indispensable for parasite virulence in laboratory inbred mice (*M. m. domesticus*). We conclude that murine resistance to *Toxoplasma* is modulated by complex interactions between host and parasite genotypes and may be independent of known effector Irgs on murine chromosome 11.

## Highlights

• Virulence in *Toxoplasma* varies with parasite strains and mouse sub-species.
• The resistance of *Mus musculus musculus* to clonal *Toxoplasma* strains is modulated by a locus on murine chromosome 11.
• *Toxoplasma* strain differences in virulence in *Mus musculus musculus* cannot be explained by individual ROP5 alleles.

## Graphical abstract

**Figure:**
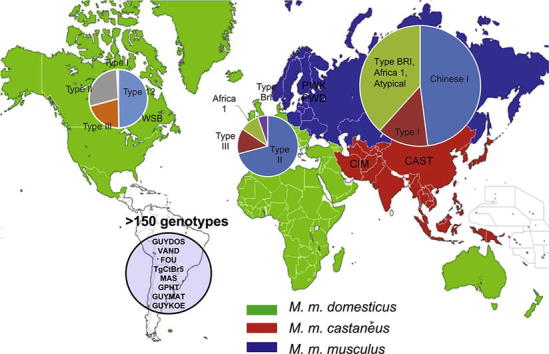

## 1 Introduction

*Toxoplasma* is a ubiquitous obligate intracellular protozoan parasite that can infect virtually any nucleated vertebrate cell. *Toxoplasma* has sexual and asexual lifecycles, with the former occurring exclusively in felines (Dubey and Frenkel, 1976), and the latter in any warm-blooded vertebrate including humans (Dubey and Jones, 2008). Although the ability to replicate in any vertebrate host has made *Toxoplasma* nearly omnipresent (Boothroyd, 2009), it exhibits distinct geographical strain distribution patterns (Lehmann et al., 2006, Shwab et al., 2014). For example, *Toxoplasma* isolates in Europe and North America mostly belong to four clonal lineages, types I, II, III and 12 (Howe and Sibley, 1995, Khan et al., 2011). In South America, at least 150 genotypes (South American or atypical strains) that cluster into haplogroups 4–15 (Minot et al., 2012, Shwab et al., 2014, Su et al., 2012), have been isolated, but none of these are clonal. Although the factors that contributed to the genetic diversity in South America are emergent, some of these atypical strains have been associated with outbreaks of severe human toxoplasmosis (Carme et al., 2002, Carme et al., 2009). Similar to Europe and North America, Africa and Asia exhibit greater parasite strain uniformity, with types II, III, and the African 1 or Chinese I strains predominating, respectively (Shwab et al., 2014).

*Toxoplasma* establishes chronic infections – characterised by the slowly dividing and encysted bradyzoite – in the central nervous system (CNS) and muscle tissues of immune-competent hosts. Predation of chronically infected hosts by felines leads to the re-entry of the parasite into the definitive host (Dubey, 2009), while ingestion of bradyzoite-contaminated food products ensures horizontal transmission in intermediate hosts. Consequently, establishing chronic infection in the intermediate host is central to the lifecycle and transmission of *Toxoplasma*. Because house mice are sympatric with, and are preyed upon by the ubiquitous domestic cat, they are considered the most important intermediate hosts in the lifecycle and transmission of *Toxoplasma*. However, *Toxoplasma* often causes death in classical inbred laboratory mice, although the time to death varies with parasite and mouse strains (Sibley and Boothroyd, 1992, Jensen et al., 2013). Indeed, differences in virulence between *Toxoplasma* strains are largely based on the survival times of infected classical laboratory inbred and outbred mice. This raises fundamental questions on the house mouse as a critical intermediate host in parasite epidemiology and genetic diversity. However, the genome of classical laboratory inbred mice is 92% of *Mus musculus domesticus* origin (Yang et al., 2007, Yang et al., 2011), which does not capture the total genetic diversity of house mice. The three major mouse sub-species, *M. m. domesticus*, *Mus musculus musculus*, and *Mus musculus castaneus*, exhibit distinct geographical distribution patterns and diverged approximately 1 million years ago (Guenet and Bonhomme, 2003). Thus, it is plausible that *Toxoplasma* strains that are virulent in *M. m. domesticus* are avirulent in the other sub-species.

Here, we investigated the virulence of representative clonal and atypical *Toxoplasma* strains in representative *M. m. domesticus* (C57BL/6J), *M. m. castaneus* (CAST/EiJ) and *M. m. domesticus* (PWK/Phj) mouse strains. We observed that the *Toxoplasma* clonal type I strain is lethal in the laboratory inbred C57BL/6J, but non-lethal in CAST/EiJ and PWK/PhJ, while most of the South American *Toxoplasma* strains tested were lethal in all the mouse strains tested in the current study. This confirms and extends a previous study of *Toxoplasma* type I strain avirulence in CIM mice (*M. m. castaneus*). Furthermore, using a chromosomal substitution mouse strain, we show that the resistance of PWK/PhJ is due to a locus on murine chromosome 11, which included a cluster of *Irg* genes that are known to encode toxoplasmacidal effector proteins in laboratory inbred mice.

## 2 Materials and methods

### 2.1 Ethics statement

All animal experiments were performed in strict accordance with the National Institutes of Health (USA), Guide for the Care and Use of Laboratory Animals and the Animal Welfare Act (USA) or were approved by the local ethical committee of the Francis Crick Institute Ltd, Mill Hill Laboratory, UK. The Massachusetts Institute of Technology (MIT; USA) Committee on Animal Care (assurance number A 3125-01) or the United Kingdom Home Office, under the Animals (Scientific Procedures) Act 1986, as a part of a project license, approved all protocols. All mice were maintained under specific pathogen-free conditions. Euthanasia was performed in a controlled CO_2_ chamber as approved by the MIT Animal Care Committee or according to Schedule 1 of the Scientific Procedures Act 1986.

### 2.2 Mice

Six to 10 week old female *M. m. domesticus* (C57BL/6J, A/J and WSB/EiJ), *M. m. castaneus* (CAST/EiJ), *M. m. musculus* (PWK/PhJ), and breeding pairs for C57BL/6J, PWK/EiJ and C57BL/6J/PWD^chr11^ consomic mice (Gregorova et al., 2008) were purchased from the Jackson Laboratories (USA). The *Unc93b1* knockout (also referred to as 3d) mice (obtained from the Ploegh laboratory, Whitehead Institute of Biomedical Research, Cambridge, MA, USA) were derived and bred in-house as previously described (Tabeta et al., 2006).

### 2.3 Parasite strains and mouse infections

*Toxoplasma* strains were maintained by serial passage on human foreskin fibroblasts (HFFs; originally obtained from the Boothroyd laboratory, Stanford University, USA). Parasites were grown in DMEM (Life Technologies, USA) supplemented with 1% fetal bovine serum (FBS; Omega Scientific, USA), 2 mM glutamine (Sigma, USA), 10 mM HEPES (pH 7.5; Sigma), and 20 µg/ml of gentamicin at 37 °C in 5% CO_2_. The following atypical strains were used (haplogroups, as defined previously (Minot et al., 2012), are shown in parentheses): CASTELLS and MAS (HG 4); GUY-KOE, GUY-MAT, and RUB (HG 5); FOU, and GPHT (HG 6); CAST (HG 7); TgCATBr5 (HG 8); GUY-DOS and VAND (HG 10). The clonal strains used in this study were the type I RH Δ*hxgprt*, RH cLUC, and GT1. The origin or genetic analysis of some of these strains has been described elsewhere (Lorenzi et al., 2016). The parasites used in mouse infection experiments were prepared by scraping T-25 flasks containing heavily vacuolated HFFs, followed by sequential passage through 25 G and 27 G needles. The released parasites were pelleted by centrifugation at 572*g* for 7 min, washed, and counted in PBS (Life Technologies). Mice were infected by i.p. injection with the required number of parasites diluted in 200 µl of PBS, and parasite viability of the inoculum was determined in a plaque assay after infections. The mice were checked twice daily and animals that became sick and progressed to severe disease were euthanised. The following were the criteria for euthanization of mice based on Body Condition Score (BCS) (Ullman-Culleré and Foltz, 1999): response to handling and/or weight loss. Mice were euthanised after (1) the inability to reach food or water for more than 24 h; (2) a 20% decrease in normal body weight (but see exception below); (3) a BCS typically less than a 2 on a 5 point scale for adult animals (but see exception below); (4) development of conditions that result in significant pain that cannot be alleviated by analgesics. With virulent strains of *Toxoplasma,* mice can die between the acute phase of infection (days 5–10) with a BCS = 3. The key indicator is lethargic response to handling. This condition can develop within hours, is highly unpredictable, and is the main reason that mice are found dead in cages. In the late acute stage of infection, days 10–15, the parasite has disseminated from the site of infection and parasitaemia is generally low throughout the body due to immune clearance mechanisms; mice can range from BCS = 3 to BCS < 2. Many mice can survive in a BCS < 2 state in the acute stages. Given the subjective nature of the BCS judgment, we have found that the best indicator for morbidity is a failure to respond to handling or extreme lethargy. Mouse weight which directly correlates with a BCS does not correlate with mortality in the early or late acute stages of infection. As mice transitioned to the chronic phase (days 15–30), mice that presented a BCS < 2 were closely monitored. For any individual infected mouse at any stage of infection (acute, transition or chronic) that entered a BCS < 2 state, that mouse was weighed and this was called the Initial BCS < 2 weight or ‘IB2 weight’. Mice that had a BCS < 2 and lost >20% from the ‘IB2 weight’ were euthanised.

### 2.4 Generation and infection of immortalised fibroblasts

Fibroblasts were generated from the diaphragm of the C57BL/6J (B6) and C57BL/6J/PWD^chr11.1^ (Chr11.1) consomic mice (Gregorova et al., 2008) as previously described (Lilue et al., 2013). The primary fibroblasts were then immortalised as previously described (Hassan et al., 2015). Wild-type RH strain parasites that ectopically express individual GUY-DOS *ROP5* alleles from a *Toxoplasma* tubulin promoter were prepared as previously described (Niedelman et al., 2012). Additionally, we made an RH parasite that expresses the wild-type or mutagenised version of the RH *ROP5C* from a tubulin promoter as described in Reese et al. (2014). ROP5C is reported to account for the virulence of the RH strain in laboratory inbred mice (Reese et al., 2014). Immortalised B6 and Chr11.1 fibroblasts were plated on cover slips and left unstimulated or stimulated with IFNG + TNF (100 ng/ml of each) for ∼ 18 h prior to infection with wild-type or the different transgenic parasites for immunity-related GTPases (Irgs) coating assays following standard procedures (Niedelman et al., 2012, Lilue et al., 2013).

### 2.5 Generation of bone marrow-derived macrophages (BMDMs) and RNA sequencing

Bone marrow-derived macrophages (BMDMs) were obtained by culturing murine marrow cells and prepared for RNA sequencing as previously described (Hassan et al., 2015). For the stimulated samples, IFNG (100 ng/ml) and TNF (100 ng/ml) were added to each well for ∼18 h. The RNA sequencing reads were mapped to the mouse genome (mm10) using Bowtie (2.0.2) (Langmead et al., 2009) and Tophat (v2.0.4) (Trapnell et al., 2009), and transcript abundance was estimated as previously described (Hassan et al., 2015).

## 3 Results

### 3.1 The clonal type I Toxoplasma strain is avirulent in *M. m. musculus* and *M. m. castaneus*

**Fig. 1:**
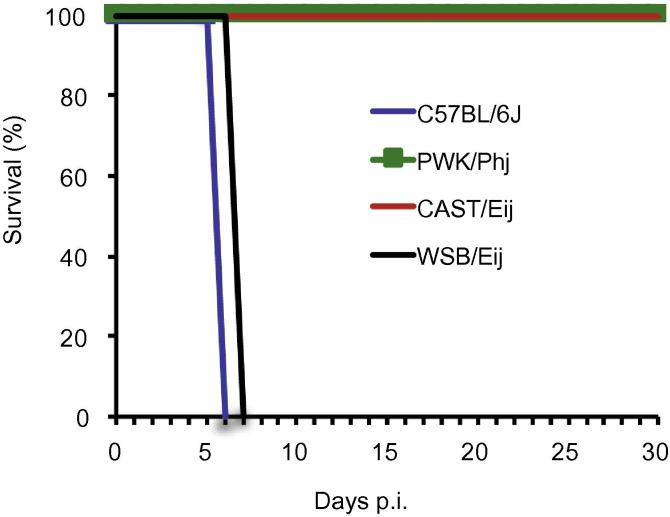
*Toxoplasma* virulence is variable among mouse sub-species. Cumulative survival data of laboratory inbred C57BL/6J (*Mus musculus domesticus*), wild-derived PWK/Phj (*Mus musculus musculus*), CAST/Eij (*Mus musculus castaneus*), and WSB/Eij (*Mus musculus domesticus*) mice i.p inoculated with 20,000 tachyzoites of a clonal type I (RH) *Toxoplasma* strain (*n* = 5 for each group).

Most clonal and atypical *Toxoplasma* strains are severely virulent in the laboratory mouse (Howe and Sibley, 1995, Saeij et al., 2006, Behnke et al., 2015), but a recent study showed that the clonal type I strain (RH), which is severely virulent (LD_100_ = 1) in classical laboratory mice, is non-lethal in wild-derived CIM mice (*M. m. castaneus*) (Lilue et al., 2013), predominant in south eastern Asia (Phifer-Rixey and Nachman, 2015). We wondered if the differences in the virulence of RH in representative *M. m. domesticus* and *M. m. castaneus* strains were due to the known genetic diversity between the mouse sub-species or was restricted to immune factors unique to the CIM mouse. Thus, instead of comparing CIM and C57BL/6J (*M. m. domesticus*) (Lilue et al., 2013), we infected different strains of *M. m. castaneus* (CAST/EiJ) and *M. m. domesticus* (WSB/EiJ) with the type I (RH) *Toxoplasma* strain*.* To explore the influence of mouse genetic diversity further, we included a representative strain of *M. m. musculus* (PWK/PhJ), which diverged from the *M. m. domesticus* and *M. m. castaneus* ∼1 million years ago. As a control, we also infected C57BL/6J mice. The RH strain was highly lethal in the C57BL/6J (B6) and WSB/Eij (WSB) mice but non-lethal in the CAST/Eij (CAST) and PWK/Phj (PWK) mouse strains (Fig. 1). In fact, we could not detect any parasites in the PWK mice using whole body imaging by the third day of infection (data not shown). Additionally, we were not able to detect parasite cysts in the brain, probably due to the reported poor cyst forming ability of the RH strain (Ferreira-da-Silva et al., 2009). The virulence of the RH strain in the representative *M. m. domestic* strains (WSB and B6), despite the WSB strain being a wild-derived strain, suggests that variable virulence of the RH *Toxoplasma* strain in these representative mouse sub-species is due to genetic diversity, as opposed to any differences between wild-derived and laboratory hybrid stocks.

### 3.2 A completely penetrant locus on chromosome 11 modulates resistance to *Toxoplasma* in *M. m. musculus*

**Fig. 2:**
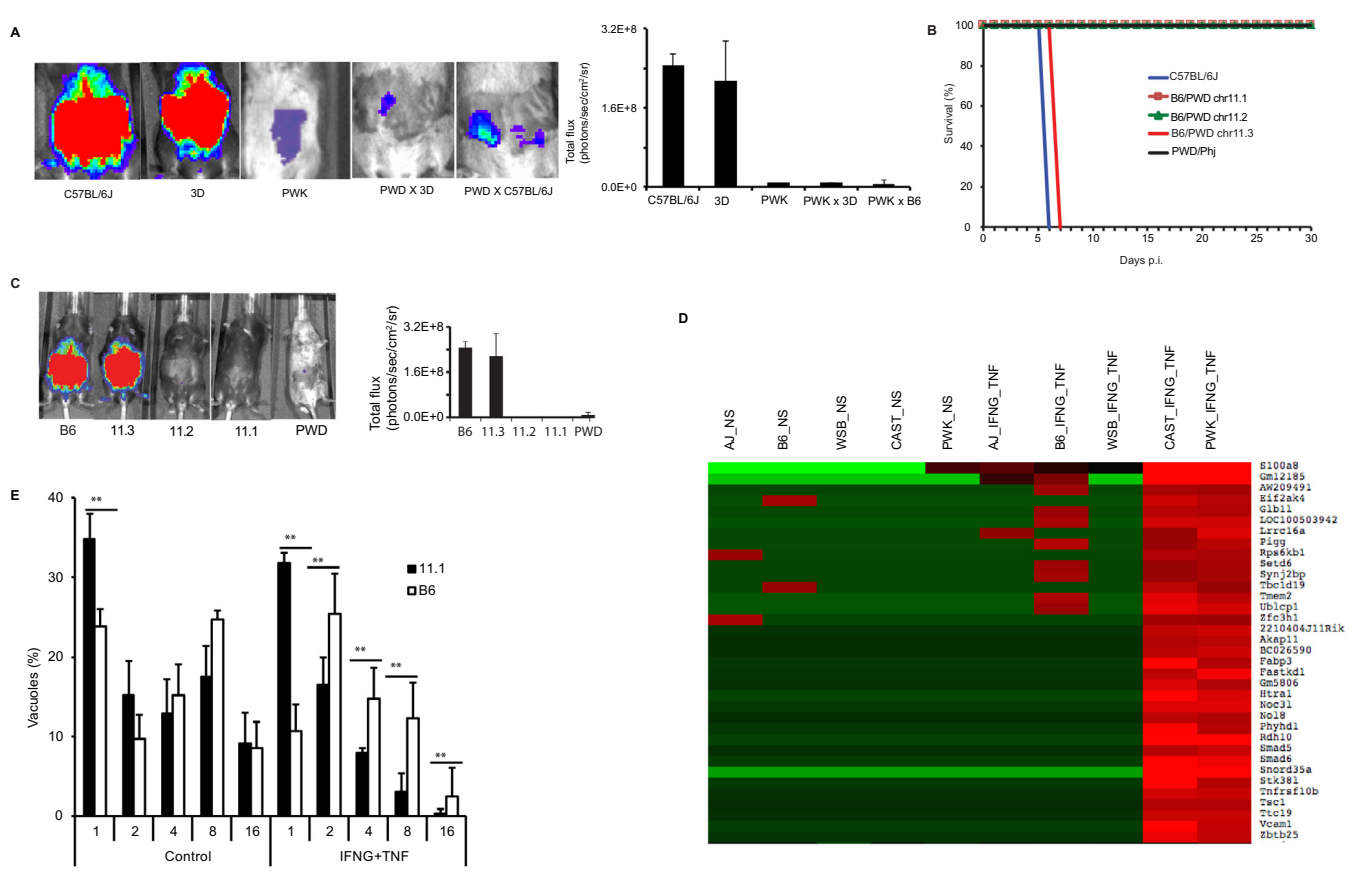
Resistance to clonal type I *Toxoplasma* strain RH in wild-derived mice is completely penetrant. (A) Representative whole body bioluminescence images and average bioluminescence (number of photons/r2) detected by whole body imaging of the parental and F1 progenies of a reciprocal cross of PWK and C57BL/6J or 3D mice on day 8 post i.p. infection with RH tachyzoites (*n* = 5 per group). (B) C57BL/6J (B6) and B6 chromosomal substitution (consomic) mouse strains carrying segments of PWD chromosome 11 (B6/PWD 11.1, B6/PWD 11.2, B6/PWD 11.3) were i.p. infected with 20,000 tachyzoites of the RH strain (*n* = 5 for each group). (C) Representative whole body bioluminescence images of B6, B6/PWD 11.1, B6/PWD 11.2, and B6/PWD 11.3 mice infected with the RH strain tachyzoites (*n* = 5 per group). (D) High throughput RNA sequencing was used to capture the transcriptional profiles of naïve or interferon gamma and tumour necrosis factor (IFNG + TNF) -stimulated bone marrow-derived macrophages (BMDMs) from different mouse strain representatives of laboratory and wild-derived mice. A representative sub-cluster of differentially expressed genes, including the tandem *Irgb2-1* (GM12185), in the *Toxoplasma*-resistant wild-derived versus the susceptible mouse strains. NS, non-stimulated BMDM. (E) Naïve or IFNG + TNF-stimulated immortalised B6/PWD 11.1 or B6 mouse fibroblasts were infected with RH strain tachyzoites and the numbers of vacuoles per field counted in three independent replicates. Mean (S.D.) is shown. ^**^P < 0.05 (Student’s *t*-test).

Besides microbial factors, optimal virulence is also dependent on host factors. The resistance of CIM (*M. m. castaneus*) mice to the RH *Toxoplasma* strain is reportedly dominant and dependent on a highly polymorphic tandem Irgb2-1 on murine chromosome 11 (Lilue et al., 2013). However, besides the *Irgs,* PWK mice carry genetic variations at several loci, including a duplication of *Unc93b1* (Keane et al., 2011). Mice carrying a loss-of-function mutation in *Unc93b1* are highly susceptible to *Toxoplasma* (Melo et al., 2010, Pifer et al., 2011, Andrade et al., 2013) while the Irgs are indispensable in murine resistance to *Toxoplasma* (Howard et al., 2011). Therefore, to elucidate the genetics of resistance to the RH *Toxoplasma* strain in *M. m. musculus*, we performed reciprocal crosses of: (i) PWK (P) and 3d (D), transgenic mice that carry a loss-of-function mutation on *Unc93b1* (Tabeta et al., 2006) (PxD/DxP); (ii) PWK and B6 (PxB/BxP); and (iii) B6 and 3d (BxD/DxB); and infected the F1 progenies with *Toxoplasma* RH strain tachyzoites. All the PWK progenies (PxD/DxP and PxB/BxP) were resistant to the RH strain, with little or no parasite burden detected from whole body imaging (Fig. 2A), unlike BxD/DxB progenies (data not shown). Therefore, resistance to *Toxoplasma* is completely dominant and independent of the duplication of the *Unc93b1* locus.

Next, we investigated whether the same locus on chromosome 11 that modulates resistance to *Toxoplasma* in CIM mice (Lilue et al., 2013) is involved in resistance to the RH strain in PWK mice (*M. m. musculus*). To do this, we utilised a B6 chromosomal substitution strain in which the chromosome 11 in B6 mice is substituted with chromosome 11 from PWD/PhJ (PWD) (B6/PWD^chr11^ consomic strain) (Gregorova et al., 2008). PWD and PWK are highly inbred mice derived from a single pair of *M. m. musculus* sub-species (Gregorova and Forejt, 2000), carry identical alleles at all loci tested, and show similar biochemical profiles (von Deimling et al., 1988). Three sub-consomic strains (B6/PWD^Chr11.1^, B6/PWD^Chr11.2^ and B6/PWD^Chr11.3^, hereafter referred to as Chr11.1, Chr11.2, and Chr11.3, respectively), carry overlapping segments of the PWD Chr11 (Gregorova et al., 2008). While PWD, Chr11.1 and Chr11.2 strains survived infection with the *Toxoplasma* RH strain, the Chr11.3 strain succumbed to infection at the same time as the laboratory inbred B6 mice (Fig. 2B). Furthermore, similar to the parental PWD mice, we could not detect the parasites in the Chr11.1 and Chr11.2 sub-consomic mice using whole body imaging (Fig. 2C). Therefore, resistance to the RH strain in PWD mice can be attributed to a locus on Chr 11 (43.8–75.4 Mb, build 36) that is shared by the Chr11.1 and Chr11.2 sub-consomic strains (Gregorova et al., 2008) and which contains, amongst other genes, the tandem *Irgb2-1*.

Although luciferase activity did not reveal mouse strain differences (PWK versus B6) in parasite burden in IFNG-stimulated BMDMs (not shown), pre-stimulation of these BMDMs with IFNG, a potent inducer of murine *Irgs*, induced high expression of *Irg2-1*, among other genes, in the BMDMs from the wild-derived mouse strains (Fig. 2D and Supplementary Table S1). However, RH parasite strain growth was significantly inhibited in IFNG-stimulated immortalised fibroblasts from the Chr11.1 mice, compared with B6 mice (Fig. 2E). Combined, these data show that, as in the CIM mice (Lilue et al., 2013), resistance to RH strain parasites in the PWK mice was modulated by a highly dominant locus on chromosome 11 (43.8–75.4 Mb), and that in fibroblasts this resistance was perhaps due to the effects of known effector *Irgs* in this locus.

### 3.3 Some atypical *Toxoplasma* strains are virulent in *M. m. musculus* and *M. m. castaneus*

**Fig. 3:**
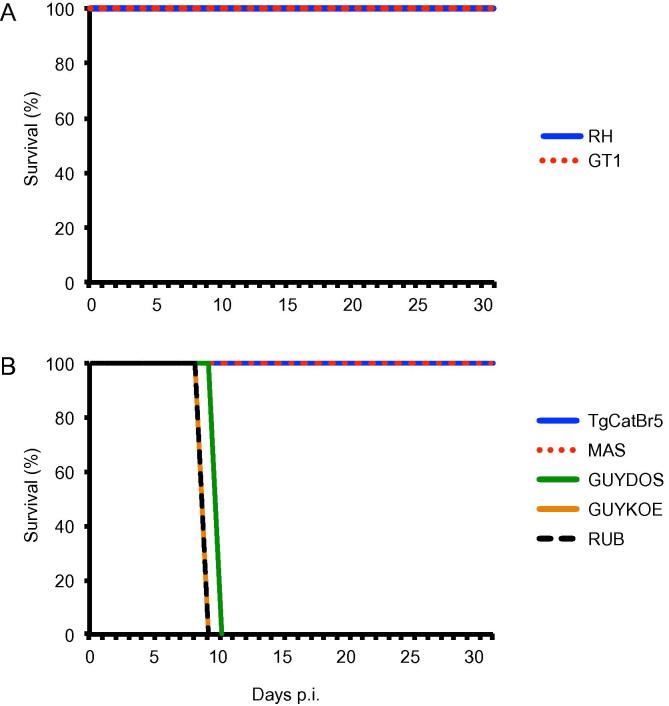
*Toxoplasma* strain differences in virulence in the wild-derived mice. Cumulative survival data of the consomic B6/PWD 11.1 mice i.p infected with 20,000 of; (A) the clonal type I and, (B) the indicated atypical strains.

Unlike Europe and North America where clonal *Toxoplasma* strains predominate (Howe and Sibley, 1995), at least 150 unique *Toxoplasma* genotypes have been isolated in South and Central America (atypical strains) (Lehmann et al., 2006, Pena et al., 2008, Minot et al., 2012). Although most atypical strains have been reported to be virulent in classical laboratory inbred mice (Dubey et al., 2013, Behnke et al., 2015), their virulence in wild-derived mouse genotypes is largely unknown. To bridge this gap in knowledge, we infected the Chr11.1 mice with different atypical *Toxoplasma* strains. As a control, we also infected these mice with the clonal type I RH and GT1 parasite strains. While the Chr11.1 mice survived infection with the clonal type I strains (Fig. 3A), they succumbed to infection with most atypical strains (Fig. 3B and Supplementary Fig. S1A). Among the virulent atypical strains, Chr11.1 survival time ranged from 8 to 18 days after infection (Fig. 3B and Supplementary Fig. S1A).

**Fig. 4:**
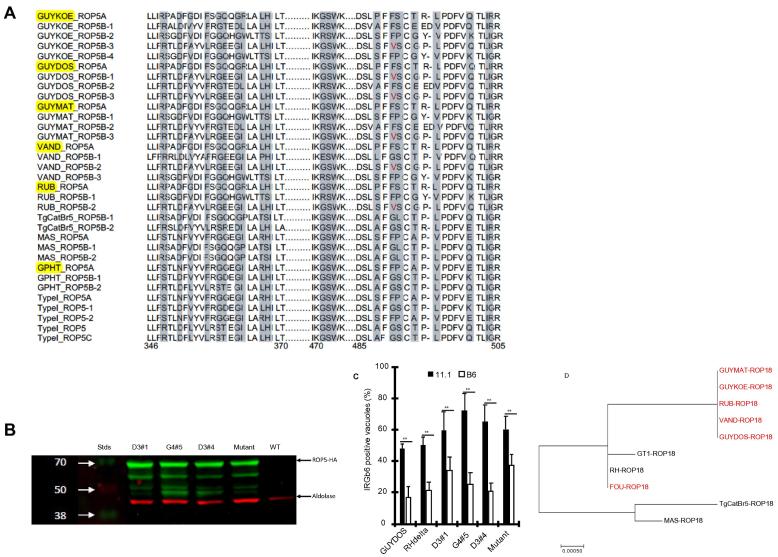
ROP5 from different *Toxoplasma* strains are highly polymorphic at regions that interact with murine IRGa6. (A) An alignment of ROP5 regions that interact with the murine Toxoplasmacidal IRGa6 (shaded grey) from different *Toxoplasma* strains. The atypical strains that are virulent in wild-derived mice are highlighted in yellow. The valine residue at position 490 that is unique to the virulent atypical strains is highlighted in red. (B) Western blot of transgenic RH ectopically expressing different C-terminally HA-tagged ROP5 alleles probed with rat anti-HA and rabbit *anti*-Aldolase. D3#1 = GUYDOS ROP5A; G4#5 = Type I ROP5C; Mutant = Type 1 ROP5C with amino acid substitution; D3#4 = GUYDOS ROP5B-3; WT = RHΔ*hxgprt*; stds = protein standards. (C) IFNG + TNF-stimulated immortalised B6/PWD 11.1 were infected with transgenic RH parasite expressing individual *ROP5* alleles and the number of parasite vacuoles coated with Irgb6 counted relative to the total number of vacuoles. (D) Molecular phylogenetic analysis of *ROP18* by Maximum Likelihood method, based on the Tamura-Nei (Tamura and Nei, 1993) model, showing trees with the highest log likelihood (−2425.6950). Red fonts indicate the atypical strains that are virulent in the wild-derived mice.

Virulence of both the atypical and clonal strains in the classical inbred laboratory mouse is reportedly due to Rhoptry protein (ROP) 5, encoded by an expanded locus of 4–10 highly polymorphic alleles (Reese et al., 2011, Niedelman et al., 2012, Behnke et al., 2015). ROP5 can bind to canonical effector Irgs and inhibit their toxoplasmacidal activity in the classical inbred laboratory mice (Fleckenstein et al., 2012; Niedelman et al., 2012). The ROP5 amino acid residues at positions 490 and 491 are reportedly essential for the allosteric interactions with Irga6 (Reese et al., 2014), an essential toxoplasmacidal effector Irg in classical laboratory inbred mice. It is postulated that in CIM mice, the tandem Irgb2-1 competitively binds ROP5, thereby freeing up the other known effector Irgs to load and vesiculate the RH strain PVM (Lilue et al., 2013). Therefore, we hypothesised that the virulent atypical strains have unique *ROP5* allele(s) that can evade sequestration by the tandem Irgb2-1 in the wild-derived mice. Although the atypical strains virulent in Chr11.1 mice in the current study form distinct *ROP5* haplogroups (Minot et al., 2012, Niedelman et al., 2012, Behnke et al., 2015), and contain multiple single nucleotide polymorphisms (SNPs) in *ROP5*, we did not identify any common SNP(s) in their *ROP5* alleles that distinguishes Chr11.1 virulent and avirulent atypical or clonal strains, except a G490V substitution in at least one allele (Fig. 4A), which is known to affect *Toxoplasma* virulence in the laboratory inbred mice (Reese et al., 2014). To test whether this substitution modulates the virulence of atypical strains in the Chr11.1 mice, we ectopically expressed individual *ROP5* alleles from a virulent atypical strain (GUYDOS) in the wild-type RH parasite strain (RH-*ROP5*_GUYDOS_). Additionally, we ectopically expressed an RH *ROP5* allele that is reported to account for RH virulence in laboratory mice (*ROP5C_I_*) (Reese et al., 2014) or a *ROP5C*_I_ that had been mutagenised to reflect the mutation G490V in the RH strain (mutant), as a control for the overexpression experiment. These represent all the known *ROP5* alleles based on the amino acid residues at positions 490 and 491 (FP, FS, GS, and VS) that form the ROP5-Irga6 interaction pocket. The ectopic expression of *ROP5* in RH strains was confirmed by western blot (Fig. 4B). Interestingly, although the Chr11.1 mice succumbed to GUYDOS, they were resistant to all the transgenic RH parasites (not shown). Next, we used the transgenic RH parasites to individually infect Chr11.1 or B6 immortalised fibroblasts pre-stimulated with IFNG, and counted the numbers of PVMs that were coated with Irgb6. We did not observe significant differences in the levels of Irgb6 coating of the RH, GUY-DOS or the transgenic RH parasite vacuoles in Chr 11.1 fibroblasts (Fig. 4C). Compared with B6 fibroblasts, we observed significantly more PVMs coated with Irgb6 in Chr11.1 fibroblasts for all the wild and transgenic parasites tested (Fig. 4C). Combined, these results suggest that, unlike in laboratory mice, the level of Irgb6 on parasite PVMs poorly correlates with virulence in the Chr11 mice.

Unlike in most clonal strains where the loss of a *Toxoplasma* kinase (ROP18) only partially affects virulence, a previous report showed a dominant role of ROP18 in the virulence of atypical *Toxoplasma* strains in a laboratory mouse strain (Behnke et al., 2015). However, a phylogenetic analysis of ROP18 from the virulent and avirulent parasites used in the current study did not cluster the parasites together based on their virulence (Fig. 4D). Neither did we observe monophyly of the virulent or avirulent strains based on ROP17 (Supplementary Fig. S1B), which is reported to contribute to *Toxoplasma* virulence in laboratory inbred mice. Thus, from the transgenic parasites and the phylogenetic analyses, the suggestion is that ROP5/17/18 do not individually explain the differences in virulence observed between atypical and clonal *Toxoplasma* strains in the wild-derived sub-species tested here.

## 4 Discussion

Chronic infection, characterised by parasite cysts in the CNS and muscle tissues, is paramount for horizontal *Toxoplasma* transmission in intermediate hosts and re-entry into the definitive feline host. Consequently, it is desirable that *Toxoplasma* achieves optimal virulence, which we define as successful infection, replication and cyst formation, without compromising the immediate survival of the host. As such, the susceptibility of classical laboratory inbred mice to *Toxoplasma* contradicts the general assumption that mice are the most important intermediate hosts in the parasite’s lifecycle*.* Since classical laboratory mice are highly inbred and exhibit only a small proportion of the genetic diversity among mouse sub-species (Keane et al., 2011), it may be that virulence of *Toxoplasma* in mice not only varies with parasite strains but also with mouse sub-species. As such, severe virulence (defined as infection resulting in the early death of the host) or avirulence (defined as a failure of the parasite to successfully colonise and replicate in the host) is only observed when a *Toxoplasma* strain infects the “wrong” mouse sub-species. In this study, we tested this hypothesis by infecting representative strains of the three major mouse sub-species with different clonal and atypical *Toxoplasma* strains. We observed that the clonal type I strains (RH and GT1), which are usually lethal in laboratory hybrid mouse stocks (*M. m. domesticus*), are avirulent in the wild-derived *M. m. musculus* and *M. m. castaneus*. On the other hand, most of the atypical strains are severely virulent in all the mouse strains tested in this study. Although we did not test many strains within the different mouse sub-species, considering the consistent virulence and avirulence of RH in the two *M. m. domesticus* (C57BL/6J and WSB/Eij) and *M. m. castaneus* strains (CIM and CAST/EiJ), respectively, it is possible that parasite virulence varies with mouse sub-species and other animals, apart from mice, are important intermediate hosts in the life cycle of some atypical strains.

The observed avirulence of the *Toxoplasma* RH strain in *M. m. castaneus* is consistent with a previous observation (Lilue et al., 2013). *Mus musculus. domesticus*, *M. m. musculus,* and *M. m. castaneus* diverged approximately 1 million years ago (Bonhomme et al., 1986) and, similar to *Toxoplasma,* have distinct geographical distribution patterns. *Mus musculus domesticus* is predominant in Western Europe, North America, and Australia. *Mus musculus castaneus* (south eastern Asian house mouse) inhabit the region from Sri Lanka, through the Indo-Malayan archipelago, to south eastern Asia, China and Japan. *Mus musculus musculus* (Eastern European house mouse) inhabits mainly the region spanning Eastern Europe through the former USSR, and northern China to Japan (Bonhomme et al., 1986). Because the *Toxoplasma* RH strain is frequently found in isolates from Asia and the atypical strains circulate mostly in South and Central America (Su et al., 2012), host–parasite co-evolution is an attractive hypothesis for *Toxoplasma* strain differences in virulence in house mice. However, it is intriguing that only a fraction of the South American *Toxoplasma* strains are severely virulent in *M. m. musculus*. Further studies including different infection models that test more parasite strains and their sympatric mouse sub-species e.g. Chinese I strains with *M. m. musculus* are needed to comprehensively test the mouse-*Toxoplasma* co-evolution hypothesis. For comprehensive analyses, such tests should be expanded to include other rodents, e.g. rodents that are more common in South America, as South American *Toxoplasma* strains might have co-evolved with South American rodents, such as the Capybara, which are also more likely prey for South American felines such as jaguars.

In the laboratory mouse, rhoptry protein (ROP) 5 is important for *Toxoplasma* strain differences in virulence (Reese et al., 2011, Niedelman et al., 2012, Behnke et al., 2015). Previously, it was reported that the resistance of a *M. m. castaneus* mouse to the clonal type I *Toxoplasma* strain was due to a tandem Irgb2-1 protein on murine chromosome 11 (Lilue et al., 2013). The hypothesis is that, in these mice, Irgb2-1 binds tightly to and sequesters ROP5, thus freeing up Irgb6, an effector Irg, to localise and destroy the PVM. Consequently, the atypical strains that are acutely lethal in the wild-derived mice have either a unique *ROP5* allele that can evade Irgb2-1 from *M. m. castaneus* and *M. m. musculus* or have extra copies of ROP5 for binding both Irgb2-1 and the other known effector Irgs. Interestingly, heterologous expression of individual *ROP5* alleles from GUYDOS (a virulent atypical strain) did not alter the virulence of the type I (RH) *Toxoplasma* strain in *M. m. musculus*. Additionally, compared with the avirulent clonal strains, we did not observe significant differences in the loading of Irgb6 to the PVM of the virulent atypical strains, which is consistent with a previous observation in laboratory mouse embryonic fibroblasts (MEF) (Niedelman et al., 2012). Although the temptation is to perturb the entire *ROP5* locus in the virulent atypical strains (Behnke et al., 2015) and measure virulence in the wild-derived mice, we did not consider this option since *ROP5* knockout parasites are generally avirulent in all mouse strains tested in independent studies. However, in light of the current results, it may be necessary, in the future, to knockout the entire ROP5 locus in a virulent atypical strain, e.g. GUYDOS, and test its virulence in *M. m. musculus* or *M. m. castaneus.* Alternatively, it would be informative to knock out the entire ROP5 locus in RH strain parasites and complement the knockout parasite with individual or combinations of *ROP5* alleles from the virulent atypical strains such as GUYDOS.

The loss of *ROP18* completely abrogates virulence of the atypical strains, but not the clonal type I RH *Toxoplasma* strain, in the laboratory mouse (Behnke et al., 2015), suggesting a more pronounced role for ROP18 in these parasites. However, a phylogenetic analysis of *ROP18* failed to cluster these parasites by virulence. Even though we did not perturb *ROP18* in these parasites, it is noteworthy that the atypical FOU and VAND strains, which are virulent in *M. m. musculus* mice, have different *ROP18* alleles (Khan et al., 2009). Additionally, the atypical VAND and TgCATBr5 strains are virulent and avirulent, respectively, in the wild-derive mice, yet both of these strains are virulent in the laboratory inbred mice due to ROP18 and ROP5 (Behnke et al., 2015). Furthermore, survival time was variable in mice infected with the virulent atypical strains, suggesting that virulence in these parasites is modulated by multiple loci, which would be consistent with a previous conclusion based on the infection of laboratory mice with some of these atypical strains (Khan et al., 2014). Because we did not detect cysts in these mice, probably due to the reported poor cyst-forming ability of the laboratory adapted RH strain (Ferreira-da-Silva et al., 2009), a future study with a more robust cyst-forming type I strain will reveal whether the type I strains are optimally adapted to the PWK mice. Combined, the current study suggests that: (i) different effector proteins, not ROP5, regulate *Toxoplasma* strain differences in virulence in *M. m. musculus*; (ii) the nature of the ROP5-Irga6 interaction is different in *M. m. musculus* versus laboratory mice, potentially involving different amino acid residues. It is noteworthy that the absence of a correlation between Irgb6 coating of PVMs with virulence in the current study is consistent with the observation in the atypical COUGAR strain, which is severely virulent in laboratory mice (Khan et al., 2009) but is as highly coated with Irgb6 as the avirulent type II clonal strain in MEFs (Niedelman et al., 2012). Thus, in some instances, the level of canonical Irg loading to the PVM is not predictive of virulence. However, because a locus on murine chromosome 11, which includes the cluster of *Irgs*, regulates resistance to *Toxoplasma*, it is possible that a non-canonical Irg or other non-Irg protein on this locus regulates the observed resistance of these mice to *Toxoplasma*. Because *ROP5/17/18* work in concert to regulate parasite virulence in the laboratory mice (Etheridge et al., 2014) it is possible that, although we generated transgenic RH parasites with the desired GUYDOS *ROP5*, we could not recapitulate GUYDOS virulence due to the absence of the virulent *ROP17/18* in the RH background. Therefore, in the future, using the Chr11.1 sub-consomic strain, which is identical to the susceptible C57BL/6J mice except for the small PWD chromosome 11 fragments, in conjunction with transgenic RHΔ*rop5/17/18* strains that have been complemented with individual *ROP5/17/18* alleles from any of the virulent atypical strains, or a genetic cross of the clonal type I and GUYDOS strains, might reveal the role of Irg-ROP interaction in disease pathogenesis of atypical *Toxoplasma* strains.

## Appendix A Supplementary data

Supplementary Fig. S1 Additional South American (atypical) *Toxoplasma* strains (GUY-MAT, VAND, FOU, and GPHT) exhibit variable virulence in consomic mice. (A) Survival times of B6/PWD 11.1 consomic mice infected with different atypical *Toxoplasma* strains. (B) Molecular phylogenetic analysis of ROP17 in different *Toxoplasma* strains that are virulent (VAND,RUB and, FOU) or avirulent (GT1, MAS and, ME49) in wild-derived mice (PWD/Phj) by Maximum Likelihood.

Supplementary Table S1 Transcript abundance, represented as fragments per kilobase exon per million reads (FPKM), in naïve and interferon gamma (IFNG)-stimulated bone-derived macrophages (BMDMs) from A/J, C57BL/6J, PWK/Phj, CAST/Eij, and WSB/Eij mouse strains. TNF, tumour necrosis factor alpha.
