# Supplementary figures and images for: Clonal and atypical Toxoplasma strain differences in virulence vary with mouse sub-species
Source: Int J Parasitol. 2019 Jan;49(1):63–70. doi: 10.1016/j.ijpara.2018.08.007 (PMC6344230; doi:10.1016/j.ijpara.2018.08.007)

A

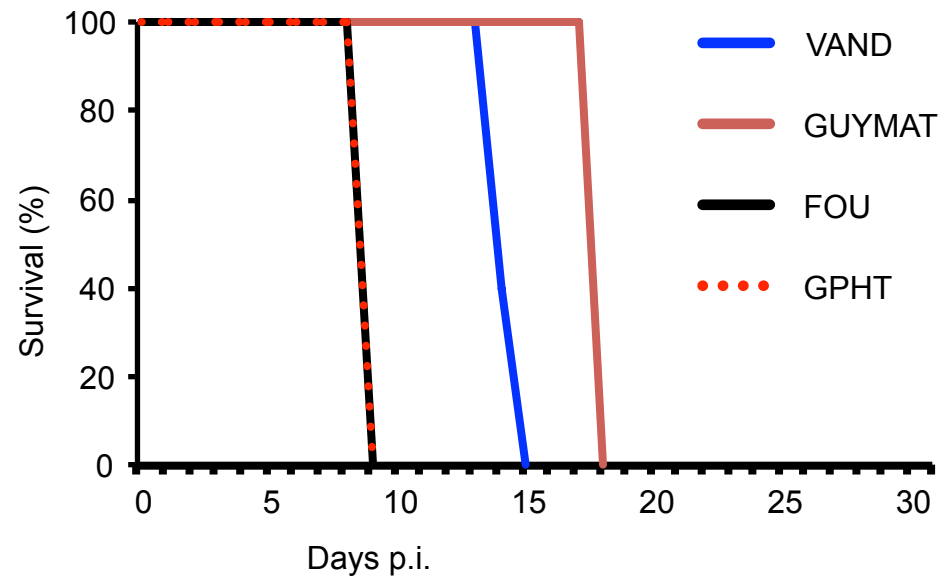

B

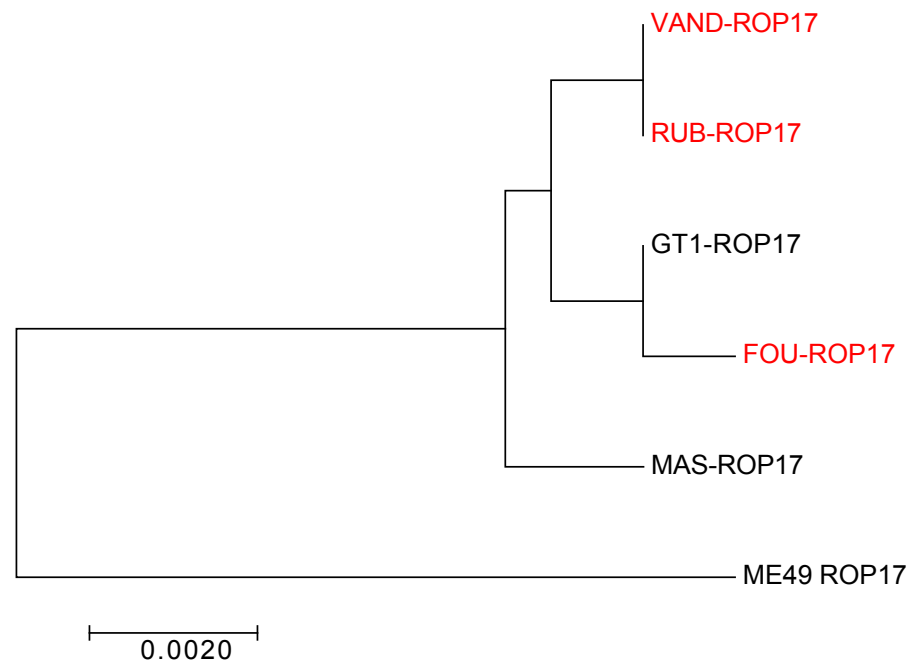

Supplement: Supplementary Table S1 — Transcript abundance, represented as fragments per kilobase exon per million reads (FPKM), in naïve and interferon gamma (IFNG)-stimulated bone-derived macrophages (BMDMs) from A/J, C57BL/6J, PWK/Phj, CAST/Eij, and WSB/Eij mouse strains. TNF, tumour necrosis factor alpha. [file mmc2.pdf]
